# Development and Validation of Filial Piety Representations at Parents’ End of Life Scale

**DOI:** 10.3390/healthcare10061054

**Published:** 2022-06-06

**Authors:** Sok-Leng Che, Wai-I Ng, Xiang Li, Mingxia Zhu

**Affiliations:** 1 Nursing and Health Education Research Centre, Kiang Wu Nursing College of Macau, Macao SAR 999078, China; shirley@kwnc.edu.mo; 2 Education Department, Kiang Wu Nursing College of Macau, Macao SAR 999078, China; shirleyli@kwnc.edu.mo (X.L.); zmx@kwnc.edu.mo (M.Z.)

**Keywords:** filial piety, end-of-life care, scale development, Macao Chinese

## Abstract

Filial piety has a long historical standing in Chinese communities. However, the filial piety practices of adult children at the end of a parent’s life are under-explored. This study aims to develop a measurement for filial piety representations of the adult children of Macao Chinese, whose parents are at the stage of end of life. By adopting a scale development and validation framework, a 19-item Filial Piety Representations at Parents’ End of Life Scale (FPR-EoL) was formulated based on a Dual Filial Piety Model and literature, through procedures of item identification, panel review, cognitive interviews, and pre-test. The FPR-EoL was examined on 274 individuals. Factor analysis showed four factors in the scale; respect and comfort, acceptance of death, spending final days, and disclosing bad news. The Cronbach’s alpha of FPR-EoL was 0.73, and the four factors were 0.73, 0.66, 0.58 and 0.77, respectively. Discriminant validity was examined between FPR-EoL, the Good Death Inventory (GDI) and the Filial Piety Scale (FPS). The results suggested that there were differences between the three scales. FPR-EoL is found to be a reliable, valid and novel measure of filial piety representations among Macao Chinese. It may be a potential tool to probe and achieve good death among older persons of Chinese ethnicity in clinical settings.

## 1. Introduction

There has been a long practice of filial piety in Chinese societies, and it is seen as the most emphasised behaviour. Filial piety influences family values, relationships and interactions between nuclear and extended family members; it is also considered one of the most important indicators for intergenerational relationships, and could potentially influence the quality of life and way of care offered to the older person in the family [1,2]. It requires material and emotional demands from children to parents, such as compliance, support, honour, respect and love [3,4]. Filial piety is a multidimensional concept that is comprised of psychological, emotional and behavioural aspects [5,6,7]. Most researchers analyse filial piety through attitudes and behaviours. For instance, Chen et al. [8] used values, social beliefs, self-construal and filial piety attitudes to predict filial piety behaviours, while Wang et al. [9] explored the expectation of filial piety of older adults. Yeh [3] suggested that there are two dimensions of filial piety belief (Dual Filial Piety Model, DFPM), namely reciprocal and authoritarian filial piety, and accordingly formulated the Filial Piety Scale (FPS) [4]. Reciprocal filial piety is the adoration, emotional and pragmatic obligations that children offer to parents, in order to thank parents for their nurturing and upbringing, while authoritarian filial piety stems from a Confucian view of an individual who should respect others with higher social position no matter how the individual is treated; it reflects children’s obedience to parental authority for the request of a social role [7].

The concept of good death has been evolving with different social-cultural values and increasing medicalisation in society, and has been considered as a death with dignity [10]. While healthcare professionals consider communication with patients and family members an important element of good death [11,12], patients regard maintaining a good relationship with family members as a key contributor to good death [13,14], which can also be seen in East Asian societies [15,16]. However, under the filial norms, adult children play an important role in shouldering care for their parents [17], especially during the end of life of their parents. End-of-life decisions, namely prognosis disclosure, treatment decisions, palliative care adoption etc., are often affected by filial piety attitudes [18,19,20]. Since death is a taboo that is hardly talked about in Chinese families, discussing death is considered a curse to the parents [21,22]. Due to the belief that death cannot be talked about, children with more traditional view often tend to do everything in order to extend parents’ lives when facing parents’ dying, and consider this as “filial”, in order to fulfil the psychological need of “try one’s best then feel no guilt” [23].

In clinical practice, extended family members would see “children have done everything to extend parents’ life” as the indicator of filial piety practice [24,25]; combined with the culture of not talking about death, results in futile treatment with no decision making of the patient. Healthcare professionals have a vital role in patients’ end-of-life decision making, yet the function of the role may be compromised—collective decision-making overrides individual decision-making in Chinese familial ethics [26,27,28], rendering both patients’ “good death” and children’s filial piety practice unachievable.

Existing studies focus on measuring general filial piety attitudes in daily life, such as the Filial Piety Scale (FPS) [3], the 10-Item Contemporary Filial Piety Scale (CFPS-10) [5], and the Filial Piety Scale for Chinese Elders (FPSCE) [29]. However, there is a lack of such tools that assess filial piety representations of adult children whose parents are at the stage of end of life. Furthermore, although Chinese in Macao may share some cultural differences because of the past colonial history, Macao still embraces a deep traditional Chinese culture and Macao Chinese could probably share many similarities in view of filial piety with Mainland Chinese, as people of both places have considerably frequent communication and exchange. Therefore, this study initiated to develop an assessment tool for measuring filial piety representations of adult children when their parents are at the stage of end of life for Macao Chinese, and it is a potential clinical tool that could help to measure and achieve good death among older persons of Chinese ethnicity.

## 2. Materials and Methods

The current study adapted the framework of Boateng et al. [30] for the process of scale development and validation, which includes three phases and nine steps. The three phases include item development, scale development and scale evaluation.

### 2.1. Item Development

This step is to identify domains and items of the scale. Due to the lack of measurement of filial piety during parents’ end of life, the research team decided to combine the concepts of filial piety and good death. Referencing existing measurements of filial piety behaviours and end-of-life-related measures exclusively for the Chinese population [5,29,31,32], and the six elements of good death, which are pain and symptom management, clear decision making, preparation for death, completion, contributing to others, affirmation of the whole person [33], 43 representations were identified. The 43 items were further reduced to 23 based on content similarity, which was through consensus among the authors, and were rephrased according to the two dimensions of the Dual Filial Piety Model (DFPM) [3,4,34,35], the draft of the Filial Piety Representations at Parents’ End of Life Scale (FPR-EoL) was therefore formed.

After the identification of items, the team invited an expert panel to review the draft of the scale. Seven experts, including a senior medical physician with a background in do-not-resuscitate training for healthcare professionals, a palliative care specialised nurse, a head nurse of an intensive care unit, an ethicist, a journalist with background in advanced directive promotion, a social worker and a deputy director of social care institute, and a psychologist, were invited to review the scale items. This study used the content validity index (CVI) as the criterion for item revision or elimination [36,37]. The scale content validity index (S-CVI) and item content validity index (I-CVI) were calculated. The experts scored 1 to 4 (completely irrelevant to completely relevant) according to the relevance of items to the scale, and gave recommendations for item revision. Items with an I-CVI lower than 0.8 suggested the need to be revised or eliminated [36,37]. The research team also modified the content of the items according to experts’ opinions. There were 22 items in the scale after modification.

### 2.2. Scale Development

In order to assess the understanding of the items in the general population and to ensure the alignment of clarity, reading level and comprehension between the general population with the research team, two rounds of cognitive interviews were conducted [30]. The two rounds of cognitive interview were conducted in August 2020, 10 interviewees were recruited from different backgrounds and age groups for each round. Participants were asked to verbally express their understanding of each item of the scale and to identify the phrases or terms that are likely to lead to ambiguity. The items were modified before the second round of cognitive interview according to the suggestions from the first round. The draft of the scale was formed after the second round of cognitive interviews. All 22 items were close-ended questions with responses of “definitely will not do”, “tend not to do”, “don’t know”, “tend to do” and “definitely will do”, which were scored 1 to 5.

The 22-item draft scale was pre-tested through an online platform and face-to-face interviews from October to December 2020. There were 208 participants, the average time to administer was 13 min. The reliability of the scale in pre-test was with a Cronbach’s *α* of 0.80. Item analysis was conducted, using an item discrimination test and item-total correlations, to ensure conciseness of the scale. The item discrimination test was examined by using the 27th and 73rd percentiles of the overall score of the scale; participants with higher scores were assigned to the upper group (27th percentile), participants with lower score were assigned to the lower group (73rd percentile), then t-tests were performed on each item to test whether it has discriminative properties [38]. Since the score of the items was continual, corrected item–total correlations were used to examine the relationship between each item and the overall score of the scale excluding the item itself; items with low corrected item–total correlation (*r* < 0.30) implied potential amendment. There would be a need for item re-examination if both the item discrimination test and the corrected item–total correlation suggested the need to do so. The analysis of pre-test revealed that, when participants were asked “If the doctor has diagnosed your parents with an incurable illness, and they are estimated to have less than 6 months to live, how likely would you do the following behaviours?”, most of the participants (94.7% to 97.1%) answered “tend to do” or “definitely will do” in “I would pay for the care and medical expenses of my parents, regardless of my financial situation”, “I would help my parents to fulfil their wishes or to do what they want, so that they have no regrets” and “I would say thank you to parents for my upbringing”, which suggested these three items are non-discriminating [38]. Therefore, these three items were eliminated from the scale, the other 19 items remained.

### 2.3. Data Analysis and Scale Evaluation

Raw data were coded with Microsoft Office Excel 2013, and Statistical Package for the Social Sciences Version 22 (SPSS, v22, Chicago, IL, USA) was utilised for data manipulation and subsequent analysis. The threshold for statistical significance was set to *p* < 0.05. Only respondents who completed the full questionnaire were considered valid for analyses.

In terms of scale evaluation, item analysis was conducted as in pre-test, exploratory factor analysis (EFA) was also conducted for test of dimensionality and internal consistency, discriminant validity was examined for tests of validity. Discriminant validity was examined by conducting the correlations between the overall score and factors of the FPR-EoL and Good Death Inventory (GDI), as well as the Filial Piety Scale (FPS). Since there are conceptual differences between filial piety representations at parents’ end of life, good death and filial piety, we expected that FPR-EoL would correlate more highly with FPS, which also measures filial piety, than it does with GDI.

### 2.4. Participants and Recruitment

This study recruited Macao residents aged 18 to 74, who identified themselves as Chinese and were able to give consent and understand written Chinese. Sample size was calculated with 10 participants per survey item [39], therefore, the study aimed to recruit at least 190 participants. A convenient and snowball sampling methods were applied via online advertisements and social media platforms in the study, data collection was conducted between December 2020 and January 2021. Regarding the influence of COVID-19 pandemic to data, the first case of COVID-19 in Macao was reported in January 2020. As of April 2022, there have been a total of 82 confirmed COVID cases and over 95% of cases were overseas imported cases found during quarantine while no deaths have been reported in Macao. Therefore, although the data collection period occurred during the pandemic, we believe that the influence of the pandemic would be very limited.

### 2.5. Questionnaire

An online structured questionnaire was used to collect data. The questionnaire included four sections: (1) socio-demographic background of participants, including age, gender, education level, marital status, religious beliefs, occupation and employment status, whether they have children or siblings, closeness with parents, whether parents were alive; (2) Good Death Inventory (GDI); (3) Filial Piety Scale (FPS); (4) developed Filial Piety Representations at Parents’ End of Life Scale (FPR-EoL).

GDI was developed by Miyashita et al. [40], and is widely used to evaluate the good death of a patient from the bereaved family member’s perspective [32,41,42]. The scale consists of 54 items, had an internal consistency of 0.94 and a reliability (ICC) of 0.52. This study used the Chinese version which was translated and validated in Taiwan by Tseng [32], and the research team replaced the wording “patient” with “I” since we were examining the person him/herself. The items use 7-point Likert-type scale rating from 1 (Completely disagree) to 7 (Completely agree). The overall score ranged from 54 to 378, a higher score means the participant cares more about good death.

The FPS was originally developed by Yang et al. [43] and has been tested repeatedly since then [3,4,34,35]. The scale is comprised of 16 items, including two dimensions (reciprocal and authoritarian) of filial piety. All items were rated from 1 (Not at all important) to 6 (Extremely important) and the overall score for both dimensions ranged from 8 to 48. The Cronbach’s α for the reciprocal dimension was 0.90 and 0.79 for the authoritarian. Both GDI and FPS were approved by the authors for the use in this study.

### 2.6. Ethical Consideration

Ethical approval for this study was obtained from the Research Management and Development Department of Kiang Wu Nursing College of Macau (Approval number: 2020JAN03). All participants were informed that the administration of the questionnaire was anonymous and they voluntarily consented by clicking the “agree to participate” button prior to answering the questionnaire on the online platform.

## 3. Results

### 3.1. Socio-Demographic Characteristics of Participants

The study received a total of 274 valid responses, of which most were female (81.4%), mean age was 41.6 years (*SD* = 15.0), with a range of 18 to 74 years. More than 70% had attained a bachelor’s degree or above, over half of them were married or cohabited (55.5%) and were employed (70.4%), in which most of them were working as professionals (43.8%). The majority of the participants had at least one child (58.0%) and sibling (91.2%), most of their parents were alive and they had a good relationship with their parents (see Table 1).

### 3.2. Content Validity

The S-CVI was 0.83, and I-CVIs were 0.86 to 1.0, which suggested the scale had good content validity and the items were relevant to the scale [36,37]. The research team then revised the scale items according to the suggestions from the expert panel.

### 3.3. Item Analysis

All items were positively discriminated in the item discrimination test [38]. Item–total correlations test revealed that items 3 to 6, 9, and 16 had low correlations. Therefore, all 19 items were retained for further analyses (see Table 2).

### 3.4. Exploratory Factor Analysis

To explore the factorial structure of the FPR-EoL, all 19 items were subjected to an exploratory factor analysis (EFA) with varimax rotation and principal component analysis method. Bartlett’s test of sphericity (*χ*^2^(171) = 1296.27, *p* < 0.001) and the Kaiser–Meyer–Olkin measure (KMO value = 0.77) suggested that the observed data were suitable forEFA [44,45]. Four factors with eigenvalues greater than 1.0 emerged and factor loadings were greater than 0.40 [46], accounted for cumulative variance of 50.19%.

Factor 1 (respect and comfort) was comprised of nine items (items 8 to 15, 18) which explained 16.68% of the variance with factor loadings from 0.42 to 0.64; factor 2 (acceptance of death) was comprised of four items (items 4, 6, 7, 16) which explained 12.25% of the variance with factor loadings from 0.63 to 0.72; factor 3 (spending final days) was also comprised of four items (items 1, 2, 17, 19), which explained 11.66% of the variance with factor loadings from 0.50 to 0.69; factor 4 (disclosing bad news) was comprised of two items (items 3, 5), which explained 9.60% of the variance with factor loadings from 0.82 to 0.86 (see Table 3).

### 3.5. Reliability and Validity

The overall scale had an internal consistency of 0.73, while the four subscales had an internal consistency of 0.73, 0.66, 0.58 and 0.77, respectively (Table 3). Table 4 shows that the FPR-EoL was weakly associated with overall GDI (*r* = 0.29, *p* < 0.001), and moderately associated with the two dimensions of FPS (*r* = 0.44 with reciprocal, *p* < 0.001; *r* = 0.35 with authoritarian, *p* < 0.001), which suggested that there were differences between FRP-EoL and GDI, as well as with FPS, and were with acceptable discriminant validities.

## 4. Discussion

There is not yet any scale measures the filial piety representations of adult children whose parents are at the stage of end of life. It mixes both filial piety and good death when end-of-life care is practised under Chinese culture. Thus, the research team generated a pool of 23 representations which contained the concepts of filial piety and good death from previous studies [5,29,31,32,33]. Through an expert panel and two rounds of cognitive interviews, the 22-item scale was pilot-tested. In the analysis of the pilot-test, three items, “I would pay for the care and medical expenses of my parents, regardless of my financial situation”, “I would help my parents to fulfil their wishes or to do what they want, so that they have no regrets” and “I would say thank you to my parents for my upbringing”, were excluded due to the vast majority of participants answering “tend to do” or “definitely will do”, suggesting that these three items were extremely important elements for filial piety representations of children when caring for parents who are at the end of their life.

The 19-item Filial Piety Representations at Parents’ End of Life Scale (FPR-EoL) was developed and validated in a formal survey of a general population of Macao Chinese. Item discrimination test, corrected item–total correlations, exploratory factor analysis and discriminant validity were examined. Corrected item–total correlations showed that items 3 to 6, 10, and 16 had weak correlations with the overall score of the scale, while the item discrimination test suggested that all 19 items had positive discrimination. EFA revealed four factors with acceptable internal consistencies in the overall scale and four subscales. FPR-EoL was confirmed to correlate more highly with FPS in two dimensions than it does with GDI.

According to previous studies, filial piety is often conceptualised as reciprocity and authoritative obligation. In this study, the findings suggested that Macao Chinese’ daily practice of filial piety is different from the practice at the end of parents’ life. We originally assumed that filial piety representations at parents’ end of life could be classified into two dimensions as in the DFPM. However, factor analysis suggested otherwise; the EFA of FPS in this study showed two factors, namely reciprocal and authoritarian, as the original scale. However, we were not able to identify the two dimensions in FPR-EoL, even though the items were constructed based on DFPM. Therefore, it is reasonable to believe that there are differences in filial piety representations between daily practice and at the end of a parent’s life among Macao Chinese. The four factors also constitute four stages of encountering a parent’s death, from the disclosure of prognosis when the news came (disclosing bad news), to doing lots of things to comfort dying parents and even sacrificing oneself to satisfy the dying parents (respect and comfort), then accepting the truth of parents’ dying (acceptance of death), to finally preparing oneself and parents for death (spending final days).

To explore each factor of FPR-EoL, factor 4 of FRP-EoL was not correlated with the overall score of GDI, which suggested that personal pursuit of good death is not related to the disclosure of information about parents’ deaths. Rather, it has a stronger correlation with authoritarian than reciprocal filial piety. This result corresponds with the traditional Chinese culture which regards talking about death as a taboo [21,22]. Healthcare professionals in China tend to disclose “bad news” to the family caregiver, rather than to the patient [47]. Tang’s [48] study suggested that the attitudes of family caregivers towards disclosure is crucial to caregiver burden and family communication, which may affect the quality of death of the patient. The result in this study also reflected that truth disclosure is more a behaviour of authority, rather than reciprocity. However, the role of who has the authority at the end of a parent’s life needs further exploration. According to Yeh [7], authoritarian filial piety is for an individual with a lower social position (generally the adult children) to sacrifice or repress their own needs in order to cater to an individual with a higher social position (generally the parents) or social norms. Nevertheless, the authority of parents may be lost at the stage of end of life; family members may tend to conceal “bad news” from patients, older adults in particular, and parents’ needs or choices are disregarded [27,41].

Factor 1 comprises various representations involving in-depth interaction between adult children and parents. In order to practise filial piety, adult children often involve themselves in carrying out parents’ late life care [17], and family engagement may affect the quality of end-of-life care of older adults [49]. This may reflect on the correlations of those representations with both reciprocal and authoritarian filial piety, because taking the initiative to provide such care might involve an interchanged power position in the children–parent relationship. For example, items 10 and 18 reflect the adult children complying with dying parents’ expectations and the social culture, respectively; items 13 and 14 reflect children taking the initiative to require others to follow what they believe to be filial.

Factor 2 was found to have no correlation with both dimensions of the DFPM. Acceptance of death is an important step in end-of-life care before a parent dies; it refers to actions on death and dying arrangements, and may influence one’s psychological wellbeing when considering those actions [50,51]. Although death preparation can be done at any stage of life [50,51], studies showed that it is not considered important among Chinese, or at least not before the stage of end of life [50,52]. Most participants of this study had not experienced a parent’s death, so they might not have thought of preparing for parents’ deaths, this factor therefore was not correlated with either dimension of the FPS.

Factor 3 is concerned with acknowledging older parents’ choices and accompanying them in their final days. It is noted that the internal consistency of this factor was relatively low, suggesting participants were less consistent in the four items. The variation may be due to the different nature of the two components, namely choice of place (item 17 and 19) and time (items 1 and 2) in the final days of end-of-life parents. Choice of place requires a fulfilment of parents’ wishes while choice of time denotes a concession of the adult children. The latter decision may imply a greater dilemma in achievement than the former. However, both components were identified to possess special meaning for dying parents of Chinese ethnicity. In a handful of studies that discuss place of death congruence between patients and family members, Ishikawa et al. [53] revealed that there was 47% to 66% congruence on place of death between patient and family in Japan, while 84% in China [54]. Family preference and ability to help were found to play a mediating role in the relationship between patient preference and place of death [53,55], which implies the importance of adult children’s awareness of parents’ preferences. With regard to accompanying, Cong and Silverstein [56] found that in rural China, male adult children who spend time with dying parents are seen as filial. Fulfilling such care responsibilities requires intergenerational exchange in emotional and financial support between adult children and older parents [57], although providing such care suggests that adult children have a higher power position because they are able to give [58]. Therefore, this factor correlates more with reciprocal filial piety than with authoritarian.

Considering generational differences in practicing filial piety, the research team further analysed the age difference in all factors of the FPR-EoL scale. The result only showed an age difference in factor 4. Those aged 18–34 yeas had significantly lower scores in factor 4 (*F* = 8.71, *p* < 0.001) than their older counterparts, suggesting that younger participants did not incline to withhold bad news from their parents. A Japanese study [59] explored cancer patients’ preferences of receiving bad news, it revealed that younger patients cared more about receiving medical information, explanations, and question-asking than older patients, which is consistent with our result, suggesting younger generation is more supportive of disclosing bad news to parents.

The influence of the relationship between filial beliefs and parent–child relationships has been examined in order to address the issues of population aging and family caregiving under Chinese culture [60]. Healthcare professionals, especially nurses, have the role of bridging patients, family members and the medical setting. Adult children with more representations of filial piety may lead them to correlate more with family members taking on more responsibilities and having more burden [61,62]. Therefore, extra attention to the psychological state of children and appropriate intervention might be needed.

There are some limitations to this study. First, this study employed a convenience sampling method and data were collected through a self-administered online questionnaire, which might affect the representativeness [63,64]. Second, the participants in this study were relatively educated, and the proportion of professionals is high, so it is suggested that the scale in a population with different education and occupation backgrounds should be further examined. Third, although the internal consistency of the FPR-EoL has been found to be good, test–retest assessment was not examined. The test–retest validity needs to be considered in the future. Fourth, female participants contributed disproportionately to the study data, this calls for attention with data interpretation. A study [65] in mainland China found that females practised more reciprocal filial piety behaviours, while males practised a more authoritarian filial pattern. The gender difference may have an impact on the indistinguishable nature of the two dimensions of the DFPM as we originally assumed. On the other hand, gender was not found to be an influencing factor in differentiating dual filial piety representation in our previously reported findings [66]. Accordingly, the difference between genders regarding filial piety needs to be explored further.

## 5. Conclusions

This study developed and examined a novel assessment tool for measuring filial piety representations of adult children whose parents are at the end stage of life. Although the tool requires more psychometric evaluation, FPR-EoL is reliable and valid, currently shows good psychometric properties, and the content of items is easy to understand for the general population in Macao. Overall, it serves as a potential tool that can possibly be applied clinically to probe and achieve good death among older persons of Chinese ethnicity.

## Figures and Tables

**Table 1:** Socio-demographic characteristics (*n* = 274).

Variable		*n*	%
Gender			
	Male	51	18.6
	Female	223	81.4
Age (year)			
	18–34	106	38.7
	35–54	101	36.9
	55–74	67	24.5
Education level			
	High school or below	67	24.5
	Bachelor	160	58.4
	Master’s or above	47	17.2
Marital status			
	Not married	97	35.4
	Married/cohabited	152	55.5
	Separated/divorced/widowed	25	9.1
Religious beliefs			
	None	155	56.6
	Yes	119	43.4
Occupation			
	Professional	61	22.3
	Medical (assistant) professional	59	21.5
	Technician	20	7.3
	Attendant/disciplined services/mechanic/other	53	19.3
	Not employed	81	29.6
Children			
	No children	115	42.0
	Have children	159	58.0
Siblings			
	No sibling	24	8.8
	Have sibling(s)	250	91.2
Closeness with father			
	Close	149	54.4
	Not close	117	42.7
	Neutral	8	2.9
Closeness with mother			
	Close	195	71.2
	Not close	73	26.6
	Neutral	6	2.2
Father being alive			
	No	95	34.7
	Yes	179	65.3
Mather being alive			
	No	62	22.6
	Yes	212	77.4

Note: Due to rounding, percentages may not always appear to add up to 100%.

**Table 2:** Item Analysis of the Scale (*n* = 274).

Item	Item Discrimination Test	Corrected Item-Total Correlation
I would choose to temporarily leave my job and concentrate on accompanying and caring for my parents	−7.66 ***	0.32
2. If I could not accompany my parents every day, I would inform them to seek their understanding	−7.31 ***	0.42
3. I would ask medical staff not to disclose the illness to my parents in order to protect them	−6.69 ***	0.26
4. If doctors say that the treatments are no longer able to improve parents’ condition, I would consider talking with my parents about stopping the treatments	−3.08 **	0.07
5. When discussing illness with my parents, I would only report good news rather than bad news	−6.90 ***	0.25
6. I would take the initiative to talk about death with parents	−3.26 ***	0.15
7. I would help my parents to prepare for the coming of death (e.g., encourages expression of emotions, accompany, listen to them etc.)	−6.36 ***	0.35
8. I would definitely seek medical advice from a number of doctors for my parents, believing that miracles may happen	−6.74 ***	0.31
9. I would ease physical pain and suffering of my parents, instead of allowing tolerance of pain caused by the illness and treatment, and life extension	−4.95 ***	0.24
10. I would commit to living up to my parents’ expectations, even if I am unwilling or unable to do so (e.g., getting married, having children, moving back home etc.)	−10.47 ***	0.44
11. I would try my best to appease my parents’ worries about future living of other family members	−10.84 ***	0.51
12. Even if I do not agree to parents’ instructions about funeral arrangements, I would carry out them according to their wishes	−7.37 ***	0.39
13. I would protect dignity of my parents and demand others’ respect for them	−8.79 ***	0.41
14. I demand obedience from other family members (spouses, children, siblings, etc.) to my parents	−8.18 ***	0.39
15. I would actively ask parents about their inner feelings, thoughts, and needs	−8.50 ***	0.50
16. I would sign the consent of Do Not Resuscitate (no cardiopulmonary Resuscitation) according to doctor’s opinion	−4.17 ***	0.18
17. I would let parents spend the rest of their lives at where they like	−7.55 ***	0.38
18. I would follow customs and arrange a grand funeral for parents	−6.35 ***	0.30
19. I would let parents die at a place of their choice	−8.27 ***	0.49

** *p* < 0.01; *** *p* < 0.001.

**Table 3:** Exploratory Factor Analysis of the Scale (*n* = 274).

Item	Factor Loadings				Communality
	F1 Respect and Comfort	F2 Acceptance of Death	F3 Spending Final Days	F4 Disclosing Bad News	
14. I demand obedience from other family members (spouses, children, siblings, etc.) to my parents	0.64	−0.10	0.10	0.16	0.46
13. I would protect dignity of my parents and demand others’ respect for them	0.60	0.12	0.10	0.02	0.38
10. I would commit to living up to my parents’ expectations, even if I am unwilling or unable to do so (e.g., getting married, having children, moving back home etc.)	0.60	−0.06	0.15	0.20	0.42
8. I would definitely seek medical advice from a number of doctors for my parents, believing that miracles may happen	0.56	−0.31	0.17	0.09	0.45
11. I would try my best to appease my parents’ worries about future living of other family members	0.56	0.30	0.30	−0.09	0.51
15. I would actively ask parents about their inner feelings, thoughts, and needs	0.54	0.30	0.38	−0.21	0.57
12. Even if I do not agree to parents’ instructions about funeral arrangements, I would carry out them according to their wishes	0.52	−0.06	0.24	0.09	0.35
9. I would ease physical pain and suffering of my parents, instead of allowing tolerance of pain caused by the illness and treatment, and life extension	0.46	0.35	−0.24	0.04	0.39
18. I would follow customs and arrange a grand funeral for parents	0.42	−0.29	0.32	0.07	0.37
6. I would take the initiative to talk about death with parents	0.08	0.72	−0.01	−0.26	0.59
16. I would sign the consent of Do Not Resuscitate (no cardiopulmonary Resuscitation) according to doctor’s opinion	−0.13	0.65	0.13	0.26	0.52
7. I would help my parents to prepare for the coming of death (e.g., encourages expression of emotions, accompany, listen to them etc.)	0.24	0.64	0.18	−0.23	0.55
4. If doctors say that the treatments are no longer able to improve parents’ condition, I would consider talking with my parents about stopping the treatments	−0.31	0.63	0.21	0.17	0.56
17. I would let parents spend the rest of their lives at where they like	0.15	0.11	0.69	−0.05	0.51
19. I would let parents die at a place of their choice	0.35	0.13	0.67	−0.15	0.61
1. I would choose to temporarily leave my job and concentrate on accompanying and caring for my parents	0.05	−0.02	0.66	0.12	0.45
2. If I could not accompany my parents every day, I would inform them to seek their understanding	0.24	0.20	0.50	0.15	0.36
3. I would ask medical staff not to disclose the illness to my parents in order to protect them	0.15	−0.05	0.06	0.86	0.76
5. When discussing illness with my parents, I would only report good news rather than bad news	0.19	−0.01	0.01	0.82	0.71
Eigenvalues	3.17	2.33	2.22	1.82	
% of Variance	16.68	12.25	11.66	9.60	
% of Cumulative Variance	16.68	28.93	40.59	50.19	
Cronbach’s alpha	0.73	0.66	0.58	0.77	0.73

F: Factor indicates Factor 1 to 4.

**Table 4:** Correlation of FPR-EoL with Good Death Inventory and Filial Piety Scale (*n* = 274).

Sub-Domains	FPR-EoL				
	F1	F2	F3	F4	Total
GDI Total	0.14 *	0.27 ***	0.28 ***	0.06	0.29 ***
1. Physical and psychological comfort	0.03	0.09	0.10	0.10	0.11
2. Dying in a favourite place	0.16 *	0.16 **	0.25 ***	0.03	0.24 ***
3. Maintaining hope and pleasure	0.03	0.19 **	0.11	0.12	0.16 **
4. Good relationship with medical staff	0.15 *	0.19 **	0.21 ***	0.03	0.23 ***
5. Not being a burden to others	−0.05	0.26 ***	0.08	0.09	0.12 *
6. Good relationship with family	0.19 **	0.18 **	0.26 ***	0.01	0.27 ***
7. Independence	0.01	0.05	0.06	0.25***	0.11
8. Environment comfort	0.07	0.23 ***	0.28 ***	0.07	0.22 ***
9. Being respected as an individual	0.09	0.24 ***	0.21 ***	−0.07	0.19 **
10. Life completion	0.16 **	0.16 **	0.17 **	0.01	0.21 ***
11. Receiving enough treatment	0.23 ***	−0.08	0.18 **	0.14 *	0.21 ***
12. Natural death	0.05	0.34 ***	0.19 **	0.13 *	0.26 ***
13. Preparation for death	0.21 ***	0.27 ***	0.32 ***	0.09	0.35 ***
14. Control over the future	0.07	0.20 ***	0.14 *	0.03	0.17 **
15. Unawareness of death	−0.09	0.06	0.00	−0.42 ***	−0.16 **
16. Pride and beauty	−0.15 *	−0.05	−0.05	−0.18 **	−0.18 **
17. Feeling that one’s life is worth living	0.20 ***	0.20 ***	0.19 **	0.11	0.29 ***
18. Religious and spiritual comfort	0.17 **	0.14 *	0.19 **	0.14 *	0.25 ***
FPS					
Reciprocal filial piety	0.40 ***	0.06	0.48 ***	0.15 *	0.44 ***
Authoritarian filial piety	0.32 ***	−0.07	0.19 **	0.42 ***	0.35 ***

F: Factor indicates Factor 1 to 4. GDI: Good Death Inventory; FPS: Filial Piety Scale. Figures are Pearson’s correlation coefficients. * *p* < 0.05; ** *p* < 0.01; *** *p* < 0.001.

## Data Availability

The Data presented in this study are available within the article.
